# Peatland restoration increases water storage and attenuates downstream stormflow but does not guarantee an immediate reversal of long-term ecohydrological degradation

**DOI:** 10.1038/s41598-023-40285-4

**Published:** 2023-09-22

**Authors:** Naomi Gatis, Pia Benaud, Karen Anderson, Josie Ashe, Emilie Grand-Clement, David J. Luscombe, Alan Puttock, Richard E. Brazier

**Affiliations:** 1 Geography, Faculty of Environment, Science and Economy, University of Exeter, Exeter, Devon EX4 4RJ UK; 2 Centre for Resilience in Environment, Water and Waste, University of Exeter, North Park Road, Exeter, Devon EX4 4TA UK; 3 Environment and Sustainability Institute, University of Exeter, Penryn, Cornwall TR10 9FE UK

**Keywords:** Carbon cycle, Wetlands ecology, Hydrology, Ecosystem services

## Abstract

Peatland restoration is experiencing a global upsurge as a tool to protect and provide various ecosystem services. As the range of peatland types being restored diversifies, do previous findings present overly optimistic restoration expectations? In an eroding and restored upland peatland we assessed short-term (0–4 year) effects of restoration on ecohydrological functions. Restoration significantly reduced discharge from the site, transforming peat pans into pools. These retained surface water over half the time and were deeper during wet periods than before. In the surrounding haggs water tables stabilised, as drawdown during dry conditions reduced, increasing the saturated peat thickness. Despite these changes, there were no effects on photosynthesis, ecosystem respiration or dissolved organic carbon loads leaving the site. Soil respiration did not decrease as water tables rose, but methane emissions were higher from rewet pools. Restoration has had a dramatic effect on hydrology, however, consequent changes in other ecosystem functions were not measured in the 4 years after restoration. Whilst restoration is crucial in halting the expansion of degraded peatland areas, it is vital that practitioners and policymakers advocating for restoration are realistic about the expected outcomes and timescales over which these outcomes may manifest.

## Introduction

Peatlands are the world’s largest terrestrial carbon store, estimated to hold ~ 500 ± 100 gigatonnes of carbon^1^, despite only covering 3% of the global land area. They form where waterlogged conditions restrict soil decomposition, enabling a slow build-up of partially decomposed plant material. Over thousands of years these organic rich peat soils can become several meters thick. However, roughly 10% of the worlds peatlands are degraded^2^ due to drainage for forestry or agriculture, burning, overgrazing, extraction for horticulture and climate change^3–5^, so instead of slowly drawing down and storing carbon, many of these peatlands are rapidly releasing carbon, exacerbating the current climate emergency^5,6^.

Wetland rewetting is a voluntary activity for reporting under the Kyoto Protocol^7^, consequently, governments and businesses are increasingly investing in peatland restoration through carbon trading initatives. Typically, restoration is carried out by conservation organisations, non-governmental organisations, landowners and multi-group partnerships, who have developed the necessary knowledge and practical skills. Water companies have also invested in rewetting peatlands, where they drain into the rivers and reservoirs used for drinking water supplies. Initially focused on reducing water discolouration at source and lowering the amount of chemicals and energy required to treat the water to potable standards^8^, more recently rewetting is being considered part of the natural flood management toolbox^9^.

Restoration usually consists of a physical intervention (for example, ditch blocking, bund building and reprofiling) to alter the flow of water across the landscape^10^. By slowing overland flow, water stays in the peatland longer, raising and stabilising water tables^9^. Wetter conditions should promote the re-establishment of more typical peatland communities, sometimes combined with further interventions such as *Sphagnum* planting or moss layer-transfer techniques^11^. A change in ecohydrological condition, that is a change in the hydrology and ecology and their interactions, is key to the longer-term re-establishment of a peatland’s many beneficial ecosystem functions^12^.

Initially, peatland restoration focused on cut-away peats (following peat abstraction for horticulture), and forested or large, bare, eroding gullies with variable results^10,13^. The range of peatlands and types of degradation included in restoration have recently diversified to meet the challenge of Net Zero targets, provide natural flood management and improve drinking water quality, among other functions. It is now more important than ever to accurately represent the effects across a broader range of peatland habitats, to prevent overly optimistic targets.

Herein, we quantify the short-term (0–4 year) effects of rewetting on a peatland’s multiple ecohydrological functions by combining hydrology, water quality and greenhouse gas emission data collected from a degraded and eroding, and then subsequently restored upland peatland in South West England. Restoration at this site involved moving peat, either from nearby borrow pits or small protuberances, to form hydrological barriers to disconnect dendritic erosional features and block gullies.

We hypothesised that a disruption in hydrological connectivity by peat blocks would reduce flow from the site (peak discharge and lag times)^9^; this would raise and stabilise water table depths^9,14,15^, in turn this would decrease gaseous carbon losses^14,16–18^—with any increase in methane offset by a reduction in the net ecosystem exchange of carbon dioxide^16,18^. Raised water tables would also reduce the production of dissolved and gaseous carbon by peat soil decomposition (as measured by heterotrophic respiration)^19^ reducing aquatic carbon losses^20^ thereby improving water quality.

## Results

**Figure 1:**
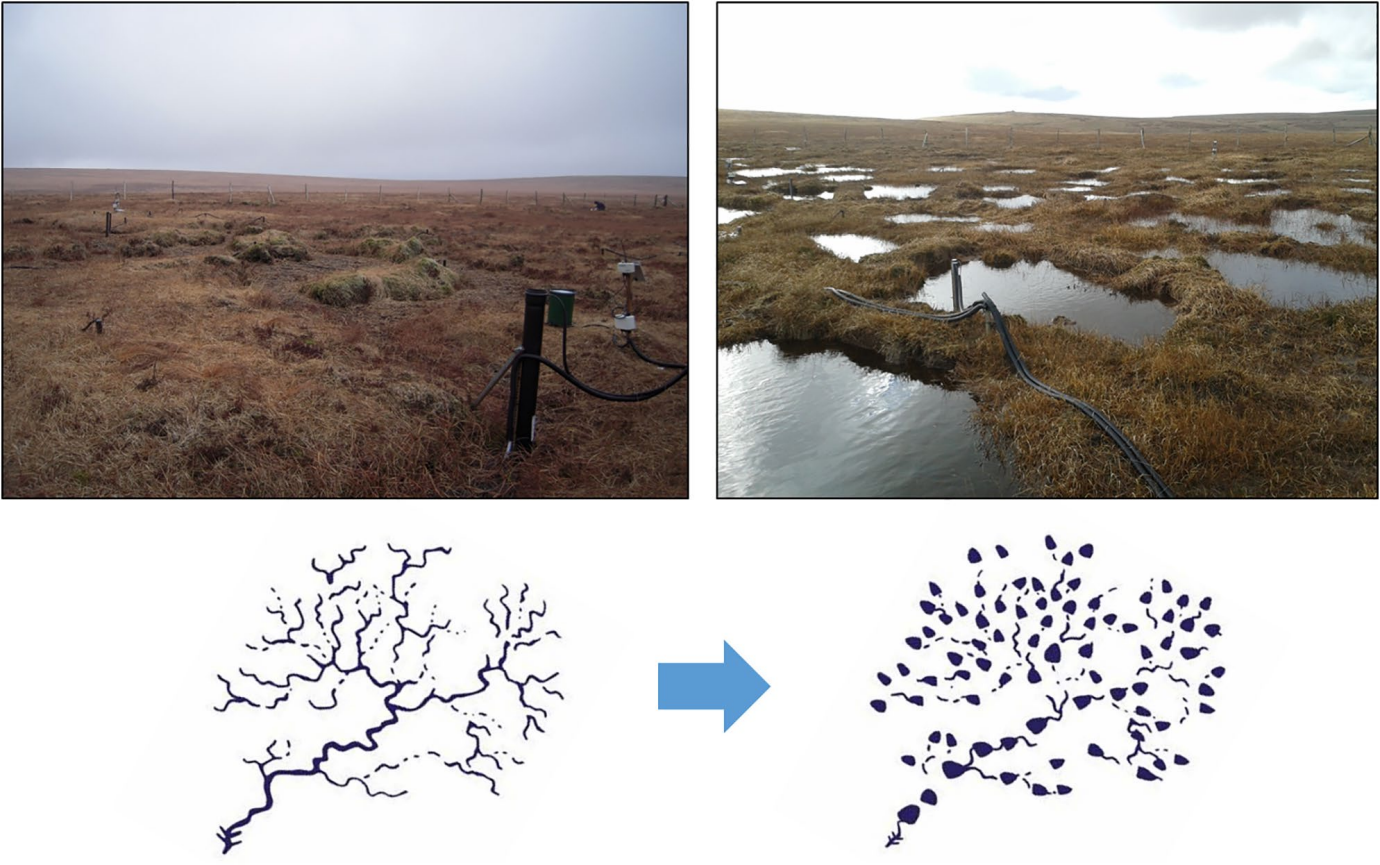
Images of the site before and after restoration with a conceptual diagram showing how rewetting has altered the hydrological connectivity of dendritic flow pathways across the site.

The action of restoration caused previously connected peat pans to be isolated by peat blocks, which physically obstructed the dendritic flow pathways, enabling the accumulation of surface water. From the first rainfall event following restoration, an increase in pooled surface water was apparent (Fig. 1). The peat pan areas were typically 1.5 m wide, < 0.5 m deep and surrounded by steep sided, vegetated peat haggs which can be seen before and after restoration in Fig. 1.

### Reduction in flow

**Figure 2:**
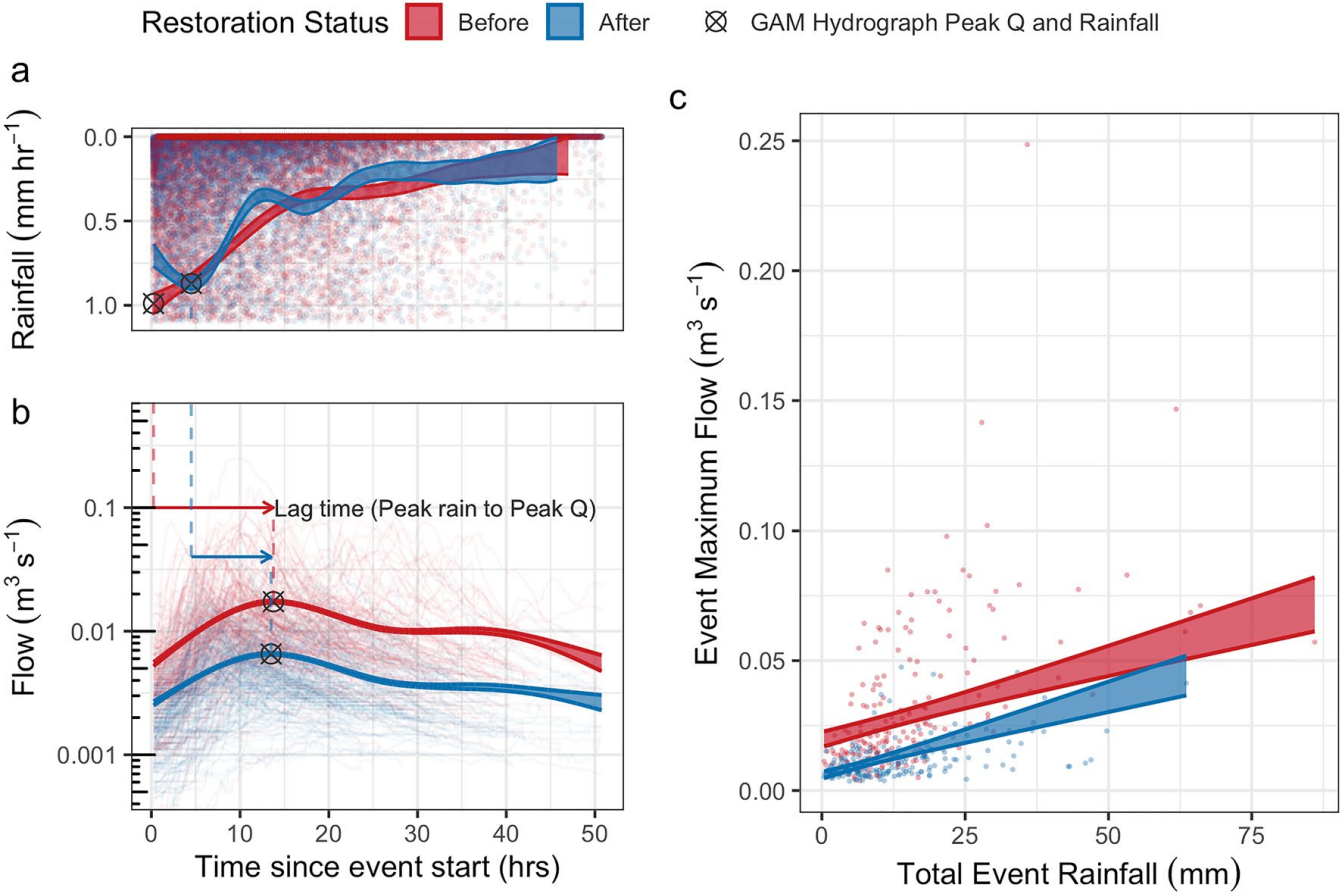
Shaded ribbons represent model 95% confidence limits for before and after restoration. General Additive Model (GAM) hydrographs with individual rainfall records presented as points (**a**) and individual event hydrographs presented as lines (**b**) with average event peaks shown as black crosses. General linear model fitted to the relationship between total hydrological event rainfall and peak event discharge (**c**).

The hydrological response within the monitored downstream gully changed following restoration, demonstrated by the change in the shape of the General Additive Model (GAM) hydrograph (Fig. 2a). While rainfall event size is the most significant (p < 0.001) positive control on peak flow, restoration had a statistically significant impact on reducing peak flow (p < 0.001) (Fig. 2b), by 0.0131 m^3^ s^−1^ per unit of rainfall.

Estimated marginal means, which provide an average value while taking into consideration rainfall, demonstrate a 49% reduction in peak flows following restoration, with mean peak flow decreasing from 0.0282 CI [0.0256, 0.0307] to 0.0143 CI [0.0130, 0.0157] m^3^ s^−1^. This is accompanied by a 21.4% reduction in the overall variability in flows (based on the ratio of flows observed at 5 and 95% of the time), with the highest flows (Q5; 5th quantile) decreasing by 66%, from 0.036 to 0.012 m^3^ s^−1^ and the lowest flows (Q95; 95th quantile) decreasing by 56.7% from 0.002 to 0.001 m^3^ s^−1^. Storm event analysis also showed a 33.3% reduction in lag times, meaning the time delay between the start of rainfall and peak discharge was shorter after restoration. There was a 68.1% CI [66.7, 69.4] reduction in the mean gradient of the hydrograph rising limb.

### Water table depths

**Figure 3:**
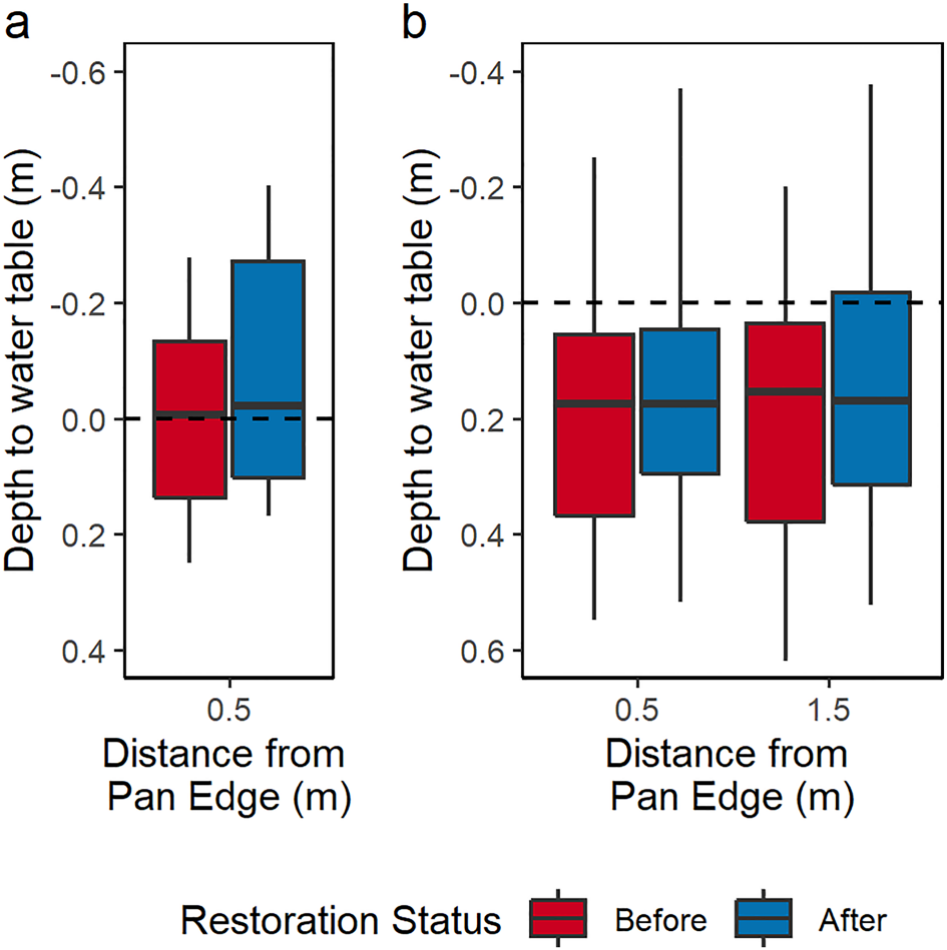
Depth to water table (m) before and after restoration in (**a**), the peat pans and (**b**), the vegetated haggs. Ground level is indicated by the horizontal dashed line, this is offset by 0.2 m between the panels as the peat pans are stepped down relative to the surrounding haggs. Vertical lines reach the minimum and maximum, boxes stretch from the 5th to the 95th quantile, the horizontal bar indicates the median.

The most striking change in the peat pans (0.5 m distance from the edge) was the increase in the 5th quantile, the water table depth achieved 5% of the time, representing wetter conditions (Fig. 3a). This increased by 0.14 m, with smaller increases occurring in the haggs at 0.5 m from the edge (0.008 m) and 1.5 m from the edge (0.053 m).

In the haggs, the most notable change was the increase in 95th quantile, the water table achieved 95% of the time, representing dry conditions (Fig. 3). This increased in all distance classes with the greatest increase 0.5 m from the edge of the peat pan (0.073 m), then 1.5 m from the edge (0.064 m) and the smallest increase occurred in the pans themselves (0.035 m).

Median water table conditions remained similar following restoration (Fig. 3) at all distances, however paired t-tests showed a significant (p < 0.001) rise in mean water tables after restoration for all distance classes. Mean water tables rose in the peat pans from 0.003 m below the ground level to 0.064 m above. In the vegetated haggs water tables rose from 0.188 to 0.175 m below the ground level in the areas closest to the edge of the pan (0.5 m) and from 0.174 to 0.161 m further away from the edge (1.5 m). Although the range increased following restoration in all distance classes (Fig. 3), the difference between the 5^th^ and 95^th^ quantiles decreased in the haggs following restoration.

### Gaseous carbon fluxes

**Figure 4:**
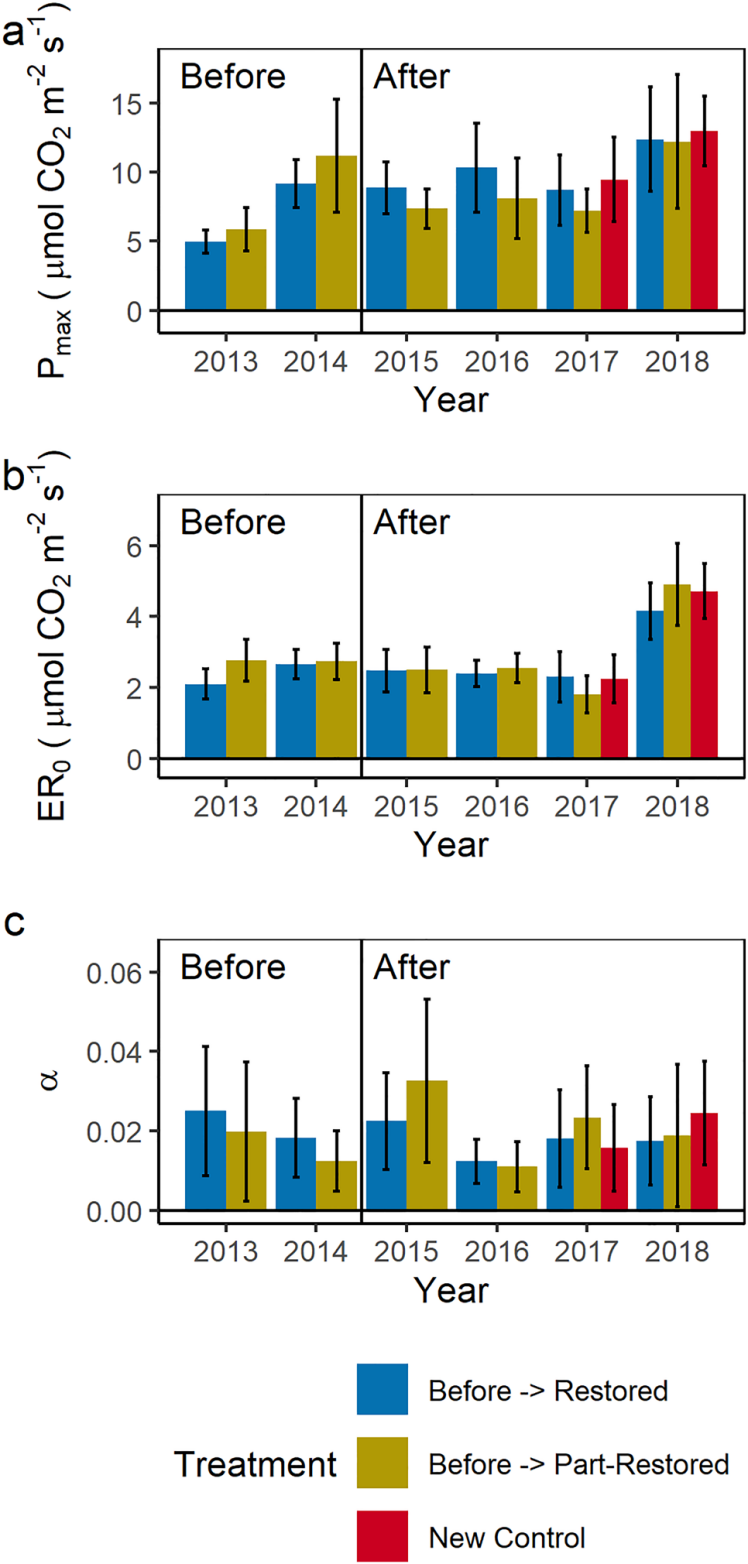
Maximum photosynthesis (**a**), ecosystem respiration (**b**) (µmol CO_2_ m^−2^ s^−1^) and alpha (**c**) from seasonal light response curves for different rewetting treatments in the vegetated haggs. Restoration occurred between the 2014 and 2015 measurements marked by the vertical line. Values are in Supplementary Information Table 1.

Restoration has had no significant impact on either photosynthesis (Fig. 4a) or ecosystem respiration (Fig. 4b). Both are highly variable between, and within, years and between monitoring locations (not shown). Ecosystem respiration ranged from 1.8 ± 0.3 to 4.9 ± 0.6 µmol CO_2_ m^−2^ s^−1^ and maximum potential photosynthesis from 4.3 ± 2.6 to 10.2 ± 2.8 µmol CO_2_ m^−2^ s^−1^). The ability to drawdown CO_2_ at low light levels, as measured by alpha^21^ (Fig. 4c), varies from 0.011 ± 0.003 to 0.33 ± 0.010, this also shows no change in response to restoration.

**Figure 5:**
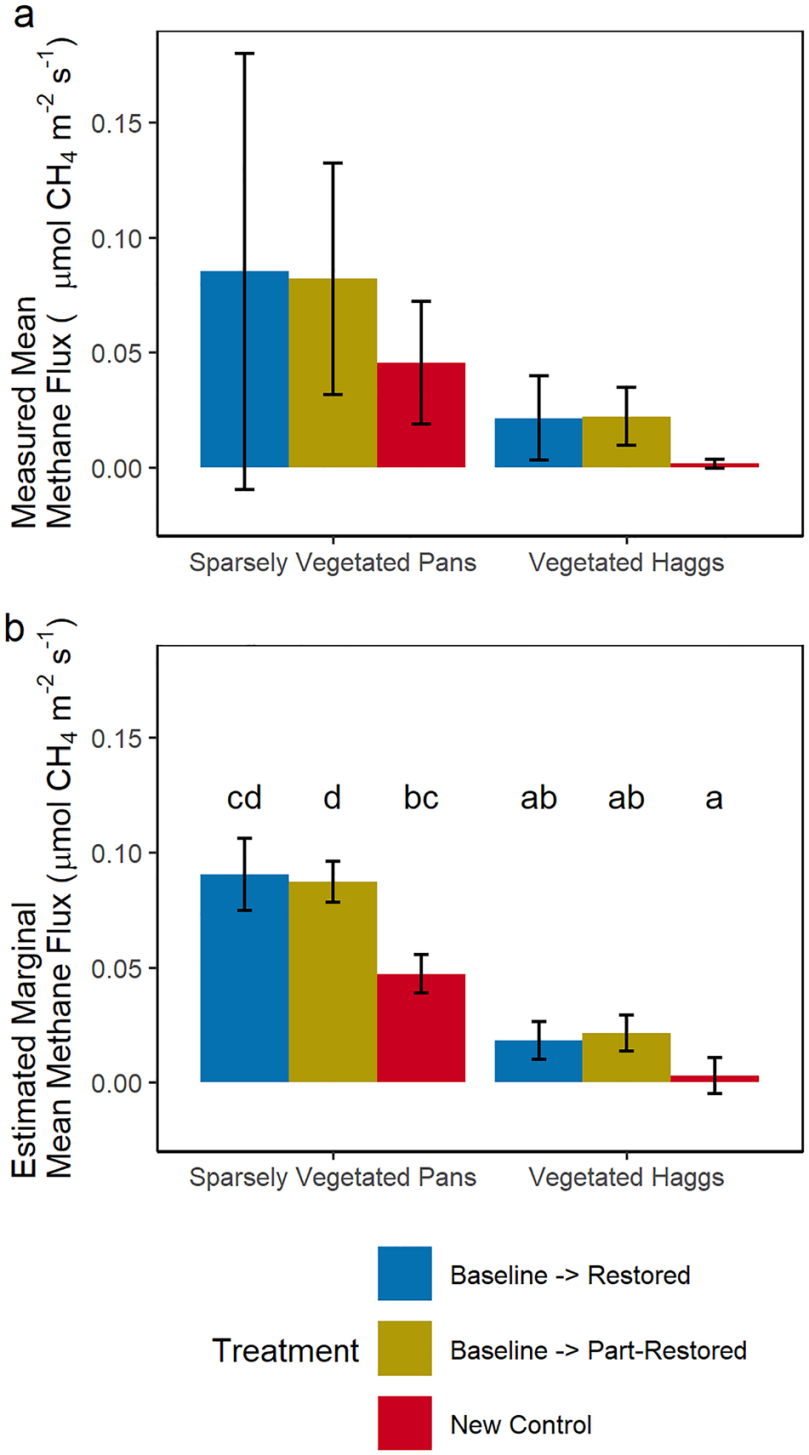
Measured mean (**a**) and estimated marginal mean (**b**) dark methane emissions (µmol CH_4_ m^−2^ s^−1^) for different rewetting treatments in vegetated haggs and sparsely vegetated peat pans. All measurements were taken following restoration. Letters denote statistically significant groups.

Measured methane fluxes were highly variable, particularly in the sparsely vegetated pans, ranging from an emission of 0.27 to a drawdown of −0.02 µmol CH_4_ m^−2^ s^−1^ (Fig. 5a). No ebullition was observed and only measurements with a linear increase in methane were included. Estimated marginal mean methane emissions were significantly (p < 0.001) greater in the sparsely vegetated peat pans than in the vegetated haggs. Allowing for the significant effects of soil temperature (p < 0.001) and spatial variation between measurement locations (Fig. 5b) there was no significant effect of rewetting in the vegetated haggs.

Estimated marginal mean methane emissions were greatest in the restored, sparsely vegetated peat pans, although this difference was not significant from the new control collars due to the large variability in measured fluxes within this group. Fluxes from the partly restored collars were significantly higher than the new control collars.

### Peat soil respiration

**Figure 6:**
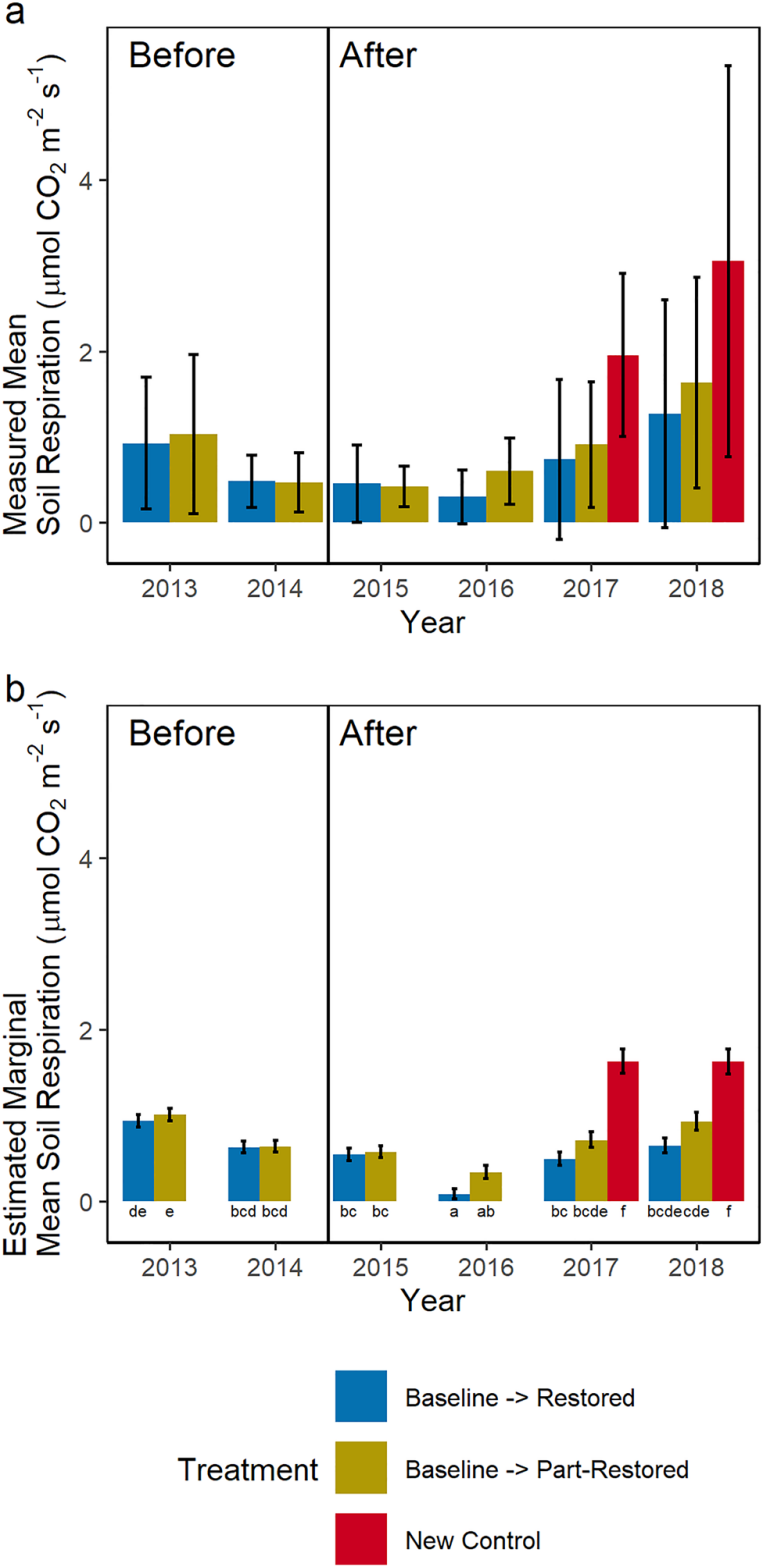
Measured mean (**a**) and estimated marginal mean (**b**) soil respiration rates (µmol CO_2_ m^−2^ s^−1^) by year for different rewetting treatments. Restoration occurred between the 2014 and 2015 measurements marked by the vertical line. Letters denote statistically significant groups.

Mean measured soil respiration was greatest in 2018 (1.3 ± 1.3 to 3.1 ± 2.3 µmol CO_2_ m^−2^ s^−1^) and least in 2016 (0.3 ± 0.3 to 0.6 ± 0.4 µmol CO_2_ m^−2^ s^−1^) (Fig. 6a) across all rewetting treatments despite restoration occurring at the end of the 2014 growing season. Allowing for the significant effects of soil temperature (p < 0.001), air temperature (p < 0.001) and spatial variation between measurement locations (Fig. 6b), estimated marginal means show significant variation between years (p < 0.001) and treatments (p < 0.001), particularly compared to the new control collars (red).

### Water quality and aqueous carbon loss

**Figure 7:**
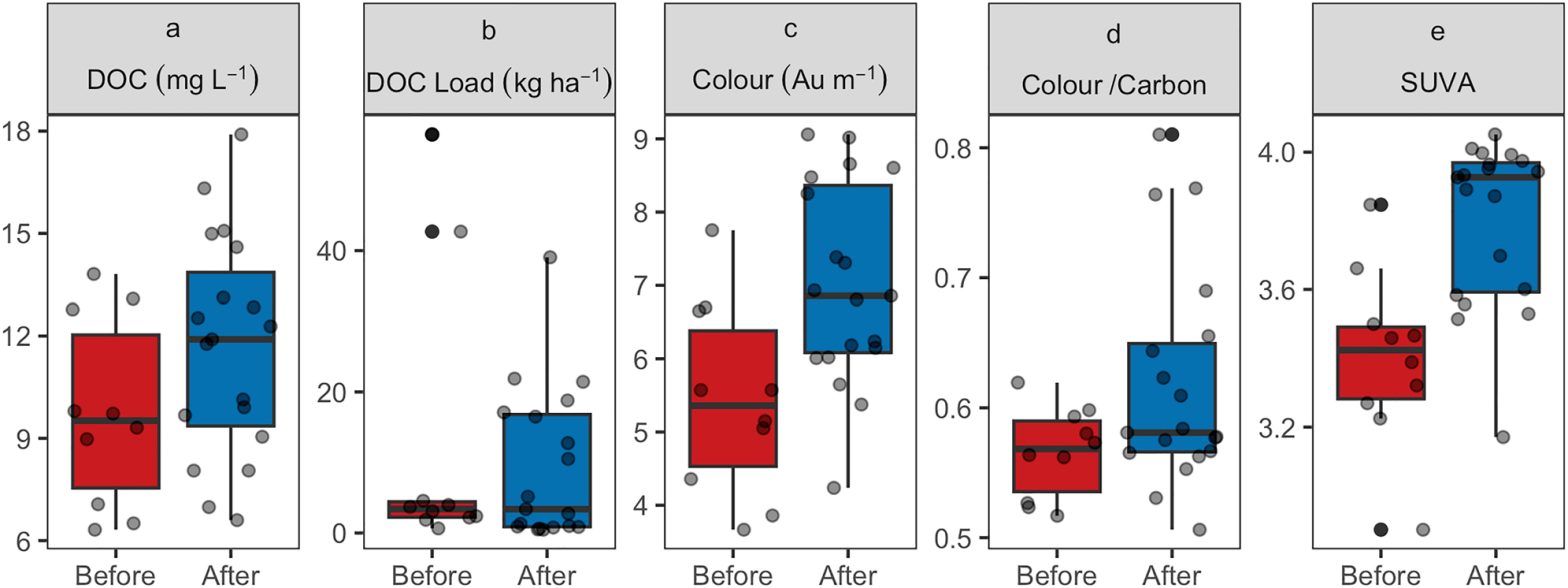
Event-based flow weighted mean concentration (FWMC) of dissolved organic carbon (DOC) (mg L^−1^) (**a**), water colour—absorption at 400 nm (Au m^−1^) (**c**), ratio of colour at 400 nm to dissolved organic carbon concentration (colour/carbon) (**d**), specific ultra-violet absorbance (SUVA)—ratio of absorption at 254 nm to dissolved organic carbon concentration (L mg^−1^ m^−1^) (**e**), and total dissolved organic carbon load per ha (kg ha^−1^) (**b**), before and after restoration.

Restoration has not had a significant positive impact on water quality or resulted in a significant reduction in aqueous carbon losses (Fig. 7). The flow weighted mean concentration (FWMC) of dissolved organic carbon (DOC) for the sampled hydrological events ranged from 6.3 to 13.8 mg L^−1^ (mean: 9.3 ± 2.8) before restoration and from 6.6 to 17.9 mg L^−1^ (mean: 12.1 ± 3.3) after restoration. This represents a non-significant increase in DOC concentrations following restoration.

**Figure 8:**
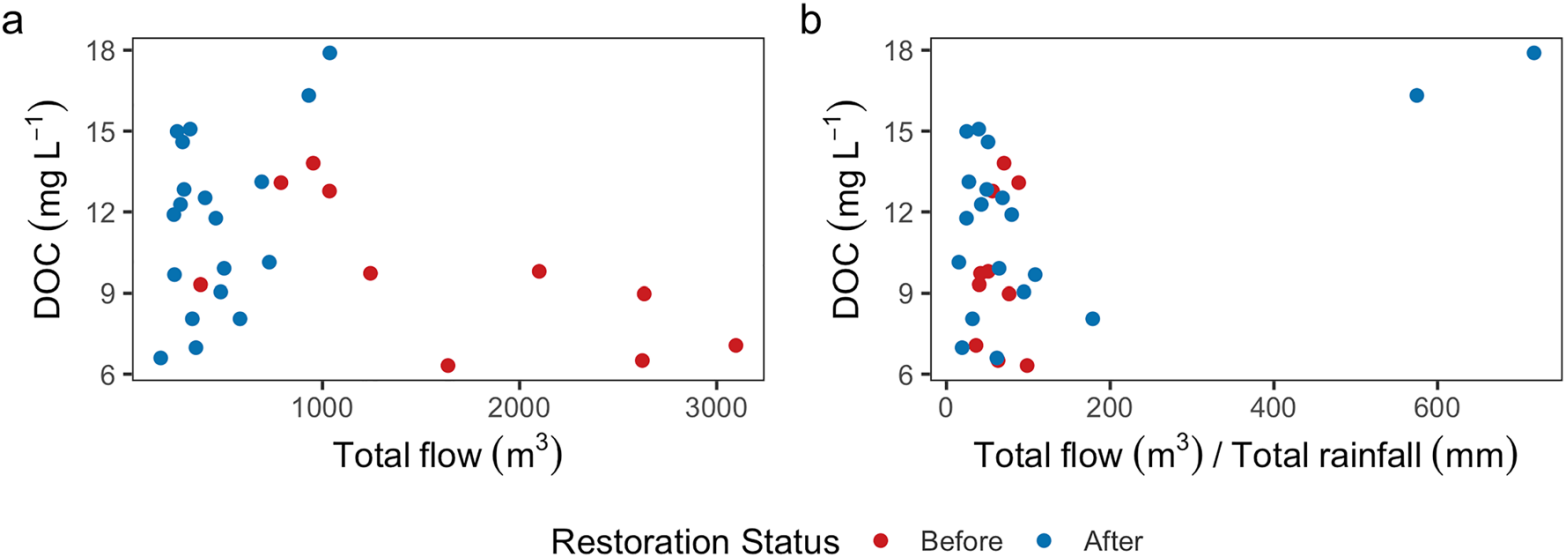
Event-based flow weighted mean concentration (FWMC) of dissolved organic carbon concentration (DOC) (mg L^−1^) against total event flow (m^3^) as measured (**a**) and normalised for rainfall (**b**) (m^3^ mm^−1^), before and after restoration.

There was also no significant correlation observed between DOC and flow, both before and after restoration (Fig. 8a). DOC concentrations were more variable with the lower flows observed after restoration, however when adjusted for total rainfall (allowing for the reduction in rainfall observed following restoration) this effect was reduced (Fig. 8b). The load of carbon exported during the monitored events varied both before (0.64 to 56.5 kg ha^−1^) and after (0.46 to 39.1 kg ha^−1^) restoration, representing the wide range of events that were sampled (Fig. 7b). However, there was no significant reduction in DOC loads resulting from restoration, with mean loads 12.2 ± 20.0 and 9.25 ± 10.8 kg ha^−1^ before and after restoration, respectively.

Restoration has resulted in a significant change to water colour (Abs^400^) (p < 0.05) and specific ultra-violet absorbance (SUVA) (p < 0.001) (Fig. 7c, e). FWMC colour, where higher DOC concentrations typically result in darker, more coloured water, increased from 5.4 ± 1.3 to 7.1 ± 1.4 Au m^−1^. There was a small, but significant increase in SUVA from 3.46 ± 0.24 to 3.76 ± 0.26 L mg^−1^ m^−1^, indicative of less hydrophilic, less labile DOC. The significant increase in colour did not result in a significant change to the colour/carbon ratio (Fig. 7d). However, the colour/carbon relationship did become more variable after restoration (from 0.57 ± 0.03 to 0.60 ± 0.08).

## Discussion

### Reduction in flow

The observed changes in the hydrograph geometry following peatland restoration provide valuable insights into hydrological dynamics (Fig. 2a). These changes indicate that restoration activities, delivering modified flow pathways and reducing hydrological connectivity^10^, have had a positive effect on the overall flow regimes within the peatland ecosystem. By physically altering the landscape, restoration has influenced overland flow pathways and the storage of water within the peat and in surface pools (Fig. 3), leading to reductions in rising limb gradient and peak flow (Fig. 2b), increasing hydrological residence time within the catchment.

The 49% reduction of peak storm flow following restoration is an important benefit of restoration (Fig. 2b). Reduced peak flows can play a crucial role in mitigating further erosion of the peatlands and minimising particulate carbon losses. This is important as organic carbon losses in solution or suspension contribute to peatland degradation, affecting their overall carbon sequestration potential, while a shift from an eroding state can lead to rapid accumulation of carbon^22^. In unrestored peatlands, the further expansion of erosional features, such as peat pans and gullies, can result in continued dissolved, particulate^23^ and gaseous carbon^24^ losses. By reducing peak flows, peatland restoration can also deliver natural flood management (NFM)^9^ by attenuating downstream flow and reducing flood risk. Incorporating peatland restoration as a component of NFM strategies at larger scales and in conjunction with other interventions within catchments^25–27^ can enhance the effectiveness of flood mitigation efforts.

Post-restoration, the lag time between the start of a rainfall event and peak discharge has decreased, aligning with the suggestion that restoration decreases water storage capacity within the peat between rainfall events^9^. A reduced lag time could also support the interpretation that flow peaks observed post-restoration were generated from the immediate area (proximal to the monitoring location), and that water further up the catchment (distal to the monitored gully) was attenuated. In theory, these changes should have a positive impact on baseflows, increasing the flow of water between rainfall events, however during this study reductions in flows during dry periods (Q95) have also been observed, consistent with the suggestion that even hydrologically intact peatlands often have minimal baseflow^28^. The implications of reduced lag times on the NFM potential of the peatland restoration, particularly during wetter months and sequential rainfall events, warrant further investigation. Most importantly, understanding the relationship between antecedent conditions, particularly recent meteorological conditions, and subsequent flow responses could provide valuable insights into how peatland restoration can contribute to water resource and flood risk management.

### Raised and stabilised water table

Mean water tables rose within the peat pans from just below the surface to above the surface following restoration with a large increase in water table depths during wet conditions (5th quantile) (Fig. 3). This occurred as peat blocks filled in gaps between adjacent haggs creating a continuous but permeable barrier within which pools could form (Fig. 1). Larger mean increases (0.22 m) have been reported behind plastic dams in gullies^14^, reflecting the greater width and depth of these gullies and the impermeable dam material but these effects will be site- and restoration-specific.

As found elsewhere^29^, the effect of restoration on mean water table depths within the haggs was small and spatially variable, driven by fine-scale topographic variation^15^. Median water table depths increased near the edge of the peat pan but reduced with increasing distance perhaps reflecting a meteorological control on water table depths. Water table depths 2 m from blocked ditches similarly showed no effect of restoration^30^. However, at both distances from the edge of the peat pan, the range in water table depths between the 5th and 95th quantiles decreased, indicating more stable water table depths following restoration. This was primarily driven by a reduction in water table drawdown during dry conditions. Similar shifts in exceedance probabilities to higher water tables during dry weather have been shown following ditch blocking^15^. The 95th quantile water table depth rose by 0.073 and 0.064 m (Fig. 3) at 0.5 and 1.5 m respectively. This represents an increase in the thickness of peat habitually saturated, which will influence the availability of oxygen in this zone and consequently effect decomposition processes.

Allowing for the offset in ground levels, 95th quantile water table depths were similar in the peat pans to the haggs. However, the change following restoration was smaller as 95^th^ quantile water table depths were closer to the surface before restoration.

### Gaseous carbon fluxes 

Peatland restoration has been found to promote carbon dioxide sequestration^16,18^, but restoration effects are most positive where restoration increases vegetation cover which increases photosynthesis. The capacity for this change is greatest where the site is bare or sparsely vegetated prior to restoration^17^. Similar to our site (Fig. 4), other vegetated drained blanket bogs have shown no significant effect of ditch blocking on CO_2_ emissions which have been attributed to high spatial and temporal variability in fluxes observed^31^ compared to the size of the restoration effect and a lack of vegetation change^32^.

The effect size was likely limited here as no significant change in vegetation was observed following restoration within the greenhouse gas monitoring areas. Transects located within the study site did show changes in vegetation^33^ but disturbance associated with monitoring may have affected vegetation growth, although *Sphagnum* growth over other equipment was widely observed.

Previously, it has been found for a *Molinia caerulea* dominated peatland, photosynthesis was higher where the water table was deeper^34^. However, the small change to median water table depths (< 0.016 m) observed in the vegetated haggs here was insufficient to either directly drive changes in photosynthesis and ecosystem respiration, which are more sensitive to normalised difference vegetation index and soil temperature at this site^24^ or to indirectly drive changes in vegetation composition away from the peat pan edges within this timeframe.

Restoration has competing effects on ecosystem respiration. Raised water tables may reduce soil respiration but increased plant cover leads to an increase in plant (autotrophic) respiration. As no changes in photosynthesis (Fig. 4a) or soil respiration (Fig. 6) were observed it is not surprising there was no change in ecosystem respiration (Fig. 4b).

Modelled growing season maximum potential photosynthesis and ecosystem respiration values were higher than annual measured values reported for a “pristine” peatland in the UK^35^, but similar to the range of modelled values found for a “pristine” ombrotrophic peatland in Canada^36,37^. However, comparisons are challenging as data for an equivalent “pristine” peatland to the one from this study could not be found.

Comparing to other peatlands with *Molinia caerulea* noted in the vegetation community, our site had higher ecosystem respiration than an Atlantic Bog in Ireland^38^, lower than a Czech drained ombrotrophic bog^39^ but similar to a temperate rewet ombrotrophic bog in Germany^40^. Maximum potential photosynthesis was similar to all three peatlands^38–40^ bearing in mind inter-annual variability.

Alpha is a measure of the ability of a plant or ecosystem to photosynthesise at lower light levels^21^, which commonly occur in these cloudy upland peatlands. Alpha values observed in this study lie within the range observed for northern peatlands^21^ and were similar to those found for a “pristine” ombrotrophic peatland in Canada^36^ but generally lower than those found for laboratory grown *Molinia caerulea*^41^, herbaceous vegetation in a rewet cut-away bog^42^ and a rewet temperate ombrotrophic bog^40^. Similar to maximum potential photosynthesis, a significant change in alpha following restoration is unlikely without vegetation change.

There are no pre-restoration methane data as portable methane analysers were not commercially available at the start of this study. Comparing across different rewetting treatments was complicated by differing initial vegetation and wetness conditions, however, methane emissions were higher in the restored and partially restored areas (Fig. 5).

Methane emissions around zero from the control vegetated haggs reflect the dryness of these areas. Whereas emissions from the rewet vegetated haggs are relatively high, reflecting a combination of higher water tables and high cotton grass (*Eriophorum *spp.) cover at these sites. Cotton grasses have hollow stems (aerenchyma)^43^ which allow methane to diffuse up the stems by-passing the oxic zone, facilitating methane emissions to the atmosphere.

Emissions from the restored peat pans lie between emissions reported for restored pools behind ditch blocks^31^ and cotton grass dominated infilled ditches^44^ reflecting the high water tables and sparse vegetation present within our pools. Emissions are significantly higher than in the control areas (Fig. 5) suggesting restoration has had an effect. As methane emissions for pristine peatlands are unknown in this area it is not possible to say if this is a short-term spike that will reduce as the cotton grasses (*Eriophorum* spp.) are succeeded by other vegetation or if emissions will continue to rise for over a decade^45^ as ecohydrological function is restored.

### Peat soil respiration

A strong link has been shown between lowering water tables and increased soil respiration^46^ as the depth to which more rapid aerobic respiration could occur increased. However, reversing this process is not as simple as raising water tables. Over time, aerated peat becomes increasingly degraded, and its physical properties change, leaving more recalcitrant, denser, less porous material behind. Rewetting does not reverse these physical changes to the peat^47^ and respiration continues to be higher from long-drained peat than less well drained peat even when water tables are similarly raised^48^. It is likely for these reasons, that despite a rise in the water table 95th quantile (Fig. 3) reducing the thickness of aerated peat, there was no trend to reducing peat soil respiration with time following restoration (Fig. 6). Respiration rates were higher in the new control sites which may indicate an effect of restoration, more likely this reflects spatial variability across the site preceding restoration.

### Water quality and aqueous carbon loss

Despite the restoration efforts and the significant changes observed in hydrological function, no significant reduction in aqueous carbon losses were observed in the 3–4 years following restoration (Fig. 7b), consistent with other UK-based studies^49,50^. Two potential processes can be considered to explain these findings. Firstly, the reductions in flow (Fig. 2b, c) following restoration may have resulted in decreased dilution of dissolved organic carbon (DOC) concentrations during rainfall events, potentially leading to higher observed DOC concentrations in the water. Alternatively, the physical changes associated with restoration activities may have temporarily increased the supply of carbon to the water, counteracting any expected reductions in carbon losses.

The significant changes in hydrological function observed after restoration, most notably the slower flows and increased water table depth, would suggest that as the site recovers from the disturbance effects of restoration, the sources of DOC should shift, resulting in fresher, lighter and more labile DOC. A positive change in DOC source would therefore result in a decrease in specific ultra-violet absorbance (SUVA) and the colour-to-carbon ratio (C/C). The slight but significant increase in SUVA (Fig. 7e) and C/C (Fig. 7d) observed in this study, suggests that while restoration activities have an effect on the source of the carbon being released, the carbon is darker and slightly less labile than before restoration. This can be attributed to short-term disturbances to the peat mass by restoration works, mobilising deeper and darker carbon into aqueous carbon pathways. However, increased DOC concentrations combined with higher aromaticity have also been found to persist following restoration in other locations^1^.

The DOC concentrations observed are lower than those reported at other degraded peatlands^50,51^ where short-term changes in DOC concentrations have also not been observed. This suggests that without an understanding of DOC exports from the same peatlands in pristine conditions, caution should be taken when relying on peatland restoration for improvements in water quality. There are examples of peatland restoration reducing DOC^20^ and it is plausible that greater time since restoration is needed before significant changes in water quality are observed on these peatlands, where decadal processes lead to increases in carbon storage^22^. Long-term studies are needed to define what realistic timescales and trajectories are for significant change of DOC sources and concentrations in response to restoration.

The variable relationship between flow and DOC concentrations observed in this study (Fig. 8a), highlight the complexities in aqueous carbon processes and pathways in degraded/restored peatlands. Instances of low DOC concentrations occurring with lower flows likely indicate the effect of supply depletion, while higher DOC concentrations in lower flows could demonstrate a reduction in dilution. To reduce uncertainty surrounding aqueous carbon processes and pathways, future studies could also incorporate continuous monitoring of DOC concentrations, capturing the effect of season and antecedent conditions. Additionally, examining hysteresis patterns, would also help elucidate the relative influence of supply depletion, dilution and spatial sources.

### Implications

The timescales of peatland restoration projects, research and monitoring projects and political cycles operate at the scale of 3–5 years whereas the ecohydrological process driving peatland communities and carbon accumulation run in decadal to centennial timescales. This mismatch makes proving positive outcomes from restoration difficult as a wider range of peatland types are studied.

High and stable water tables underpin all ecological processes in a functioning peatland. At this site restoration has had a dramatic effect on the hydrology (water tables and flow) demonstrating near immediate potential for NFM, but we are not seeing consequent changes in water quality and carbon fluxes in response to this. There are signs of progress towards more natural functioning, such as increased methane emissions, and we would expect change to continue over time.

## Methods

### Study site

The study site is in an area of degraded peatland in Dartmoor National Park (50.614° N; 3.961° W) in the south west of England. The peatland features a pattern of erosional peat pans (generally < 1 m deep) between vegetated haggs^24,52^. Where the gradient is steeper, these peat pans form dendritic erosional features and gullies. The site lies at 515 m above sea level and the vegetation is classified as National Vegetation Classification class M17 *Scirpus cespitosus–Eriophorum vaginatum* blanket mire^53^ with a high coverage of *Molinia caerulea*. Peat at the study site is estimated to be between 3.6 and 4.0 m thick^54^. Restoration occurred between August and September 2014. Peat, either from borrow pits or small protuberances, was used to block gullies and disconnect dendritic erosional features.

### Hydrological and meteorological

Stage in the gully was measured every 15 min from 09/04/2012 to 22/01/2018 using a vented submersible pressure transducer (IMSL Geo100 Impress, UK). This was converted to discharge using a rated trapezoidal flume structure and a Doppler area velocity meter (ISCO 2150, Teledyne ISCO, USA). Mean rainfall rate across the contributing area was derived from the NIMROD radar system^55^ and aggregated to 15-min timesteps. Rainfall and runoff events were identified and paired using a semi-automated rules-based approach^27^. As hydrological datasets are typically non normally distributed and event size and number varied between the before and after restoration periods, statistical approaches were adopted that account for this variability^26,27^. General Additive Models (GAM) were fitted using the gam package^56^ to explore changes in hydrograph shape and provide an approximation of mean hydrological response. As hydrological events are strongly controlled by rainfall, General Linear Models (GLM) were used to explore how peak flow were related to rainfall events, with restoration as an additive covariate, allowing changes in flow to be determined while accounting for differences in meteorological conditions.

Water table depth (WTD) was measured every 15 min using vented pressure transducers (IMSL Geo100 Impress, UK or Onset HOBO, USA) from 19/04/2012 to 28/12/2018 in a nested monitoring array in zones 0–1 m and 1–2 m from the edge of the bare peat. Dipwells were constructed from 40 mm plastic tubing, drilled with 8 mm holes, and inserted 2.5 m into the peat.

Outliers were removed by a generalised Extreme Studentized Deviate filter with a 6-h moving window, gaps up to 12 h were filled by spline interpolation. Two-sided paired t-tests were used to compare mean water table depths before and after restoration at each distance class.

### Water quality

Storm-based, flow-integrated, water sampling (up to 24 per event) was carried out from the gully using automatic pump samplers (Teledyne ISCO, USA). Samples were collected within 48 h, and subsequently stored at 4 °C in the dark prior to analysis within one week.

Filtered (0.45 μm) sub-samples were analysed by UV spectrometry for dissolved organic carbon (TriOS ProPS analyser, TriOS GmbH, Germany) and colour (at 254 nm and 400 nm), (Unicam UV4-100 analyser, Thermo-Fisher Scientific, UK) following Grand-Clement et al*.*^57^.

A selection of samples were analysed by thermal oxidation (South West Water analytics facility, Hach Lange TOC analyser, USA) or non-purgeable organic carbon analysis (TOC-VCPH Shimadzu, Japan) to build a linear regression between spectral absorbance and dissolved organic carbon. Specific ultra-violet absorbance (SUVA)^58^ and colour per carbon unit (C/C)^20^ were derived by dividing the dissolved organic carbon content by absorbance at 254 nm and 400 nm respectively.

To ensure the results captured adequately represented event conditions, event based statistics were only derived if the samples were collected within a separated event, were greater than 3 in number and covered > 70% of the event flow^57^.

The flow weighted mean concentration (FWMC) (Eq. 1) was calculated for DOC, Colour at 400 nm, SUVA and C/C to account for variations in flow and sample numbers between events, after Dinsmore et al.^59^:

1 $$FWMC= \frac{\sum ({C}_{i} \times {t}_{i}\times {Q}_{i})}{\sum ({t}_{i}\times {Q}_{i})}$$

where DOC (*FWMC*) is expressed in mg L^−1^, *C*_*i*_ is the instantaneous concentration (mg L^−1^), *t*_*i*_ is the time step between samples, and *Q*_*i*_ the instantaneous discharge (m^3^).

Total C load (kg) per event was calculated using Method5 (Eq. 2), after Walling and Webb^60^, which weighs the mean event load by the mean of all measured flow. This was calculated using the function developed for the Riverload package ^61^ and is calculated as follows:

2 $$L= K\left(\frac{\sum_{i=1}^{n} {C}_{i} \times {Q}_{i}}{\sum_{i=1}^{n}{Q}_{i}}\right)\overline{Q }$$

where *L* (g) is the total DOC load for the time period, K is a conversion factor to account for the measurement units, $\overline{Q }$ (m^3^ s^−1^) is the mean flow from the continuous record throughout the event, *Q*_*i*_ (m^3^) is the instantaneous flow, *C*_*i*_ (mg L^−1^) is the instantaneous concentration, and n the number of samples.

Following log10 transformation, all data remained non-normally distributed, so a Wilcoxon rank-sum test was used to quantify the significance of the restoration effects.

### Ecosystem gaseous carbon flux 

Net ecosystem exchange was measured in 12 locations (6 in the vegetated haggs, 6 in the erosional peat pans) before restoration and 15 locations (5 rewet vegetated, 3 control vegetated, 4 rewet peat pans and 3 control peat pans) following restoration. Measurement locations varied pre-and post-restoration as the rewetting flooded the planned control locations. Measurements were taken approximately monthly.

In the vegetated areas pre-restoration CO_2_ measurements were taken using LiCOR-8100 infra-red gas analyser (LiCOR, Lincoln, Nebraska) with an 8100-104C transparent chamber and a (LiCOR Li-190 Quantum Sensor). Post-restoration CO_2_ and CH_4_ measurements were taken using a Los Gatos Ultra-Portable Greenhouse Gas Analyser (San Jose, California, USA) with a 0.3 m diameter 0.5 m tall Perspex chamber and Quantum Sensor (Skye, Llandrindod Wells, Wales, UK).

In the peat pans pre-restoration, CO_2_ measurements were taken using an EGM-4 infra-red gas analyser and a transparent CPY-4 canopy assimilation chamber (PP Systems, Hitchin). Post-restoration CO_2_ and CH_4_ measurements were taken using a Los Gatos Ultra-Portable Greenhouse Gas Analyser (San Jose, California, USA) with a 0.3 m diameter, 0.1 m tall floating chamber. No ebullition was observed.

Pre-restoration measurements were taken on sunny days at 100, 60, 40, 10 and 0% light levels using a combination of shade cloths, post restoration only 100 and 0% light levels were measured. The chamber was removed between measurements to restore ambient conditions.

The net gas exchange was calculated from the linear change in chamber concentration measured every 2 s over 2 min. Linear accumulation rates with an r^2^ < 0.7 were discarded unless the maximum change was less than 2.8 ppm CO_2_ or ≤ 1 ppb CH_4_ in which case a 0 flux was assigned.

Hyperbolic light response curves^21^, with the formula NEE = REco – α.PPFD.Pmax/(α.PPFD + P_max_) were fitted for each growing season (June–September) for each rewetting treatment separately using nls^62^. NEE is net ecosystem exchange, Pmax the maximum rate of photosynthesis and REco the ecosystem respiration all in μmol CO_2_ m^−2^ s^−1^. PPFD is the photosynthetic photon flux density (μmol photons m^−2^ s^−1^) and α the initial slope of the rectangular hyperbola (μmol CO_2_ μmol photons^−1^).

A linear mixed effects model was fitted to dark methane emissions using lmr^63^. Soil temperature at 0.1 m, site (vegetated hagg or sparsely vegetated peat pan) and treatment group were fixed effects with an interaction. Collar code was nested within sampling date as random variables to allow for spatial and temporal heterogeneity. Although including water table depth improved the model, this was not included as this masked rewetting effects.

### Below-ground carbon dioxide respiration

Below ground soil respiration was measured within the vegetated haggs (2 replicates) at six locations pre- and post-restoration with an additional three control locations added post-restoration as the original control locations were impacted by restoration. Polyvinyl Chloride collars (0.16 m diameter, 0.08 m height) were sealed to the peat surface using non-setting putty (Evo Stick “Plumbers Mait”, Stafford, UK). The collars were routinely cleared of vegetation and trenched to 0.2 m depth in a circle 0.2 m from the collar. This excludes live roots enabling measurement of the below-ground heterotrophic component.

Soil carbon dioxide flux measurements were taken approximately fortnightly over the growing seasons before and monthly over the growing seasons after restoration using an EGM-4 infra-red gas analyser and a CPY-4 canopy assimilation chamber (PP Systems, Hitchin, UK).

A linear mixed effects model was fitted to natural logarithmic transformed respiration (LogR) rates using lmr^63^, emmeans^64^ was used to calculated the estimated marginal means for this model (LogR ~ T10 + T_Air + Year*Group + (1|LocCode)). Soil temperature (T10) at 0.1 m, chamber air temperature (T_Air), treatment group and year were fixed effects, with an interaction between treatment group and year. Collar code (LoCode) was a random variable to allow for spatial heterogeneity between locations. Although including water table depth improved the model, this was not included as this would be expected to mask rewetting effects.

## Data availability 

All data and analysis codes are available from the University of Exeter Open Research Exeter repository at 10.24378/exe.4844.

### Supplementary Information

Supplementary Table 1.
