# Supplementary material for: Peatland restoration increases water storage and attenuates downstream stormflow but does not guarantee an immediate reversal of long-term ecohydrological degradation
Source: Sci Rep. 2023 Sep 22;13:15865. doi: 10.1038/s41598-023-40285-4 (PMC10516923; doi:10.1038/s41598-023-40285-4)
Supplement: Supplementary file 1 — Supplementary Table 1. [file 41598_2023_40285_MOESM1_ESM.pdf]

Peatland restoration increases water storage and attenuates downstream stormflow but does not guarantee an immediate reversal of long-term ecohydrological degradation, Gatis, N., Benaud, P., Anderson, A., Ashe, J., Grand-Clement, E., Luscombe, D.J., Puttock, A. and Brazier, R.E.(in review) Nature Scientific Reports

Hyperbolic Light Response Curve Parameters

Year	Treatment	Restoration	n	Pmax	Pmax_SE	REco	REco_SE	alpha	alpha_SE	REco_lin	REco_lin_SE	beta	beta_SE
2013	Before -> Restored	Pre	213	5.0	0.4	2.1	0.2	0.025	0.008	1.2	0.2	0.0025	0.0002
2013	Before -> Part Restored		189	5.9	0.8	2.8	0.3	0.020	0.009	2.1	0.3	0.0029	0.0003
2014	Before -> Restored		167	9.1	0.9	2.7	0.2	0.018	0.005	2.2	0.2	0.0043	0.0002
2014	Before -> Part Restored		181	11.2	2.1	2.7	0.3	0.012	0.004	2.3	0.2	0.0044	0.0003
2015	Before -> Restored	Post	207	8.9	1.0	2.5	0.3	0.023	0.006	1.5	0.2	0.0046	0.0004
2015	Before -> Part Restored		202	7.3	0.7	2.5	0.3	0.033	0.010	1.3	0.3	0.0041	0.0004
2016	Before -> Restored		94	10.3	1.6	2.4	0.2	0.012	0.003	2.0	0.2	0.0045	0.0003
2016	Before -> Part Restored		97	8.1	1.5	2.5	0.2	0.011	0.003	2.2	0.2	0.0036	0.0003
2017	Before -> Restored		82	8.7	1.3	2.3	0.4	0.018	0.006	1.4	0.3	0.0038	0.0004
2017	Before -> Part Restored		152	7.2	0.8	1.8	0.3	0.023	0.007	0.8	0.3	0.0034	0.0004
2017	New Control		157	9.5	1.6	2.2	0.3	0.016	0.006	1.5	0.3	0.0041	0.0004
2018	Before -> Restored		80	12.4	1.9	4.2	0.4	0.018	0.006	3.4	0.4	0.0048	0.0005
2018	Before -> Part Restored		153	12.2	2.5	4.9	0.6	0.019	0.009	4.1	0.5	0.0049	0.0006
2018	New Control		153	13.0	1.3	4.7	0.4	0.024	0.007	3.7	0.4	0.0053	0.0004
